# Direct and Indirect Control of the Initiation of Meiotic Recombination by DNA Damage Checkpoint Mechanisms in Budding Yeast

**DOI:** 10.1371/journal.pone.0065875

**Published:** 2013-06-10

**Authors:** Bilge Argunhan, Sarah Farmer, Wing-Kit Leung, Yaroslav Terentyev, Neil Humphryes, Tomomi Tsubouchi, Hiroshi Toyoizumi, Hideo Tsubouchi

**Affiliations:** 1 MRC Genome Damage and Stability Centre, University of Sussex, Brighton, United Kingdom; 2 Graduate School of Accounting, Waseda University, Tokyo, Japan; 3 Department of Applied Mathematics, Waseda University, Tokyo, Japan; National Cancer Institute, United States of America

## Abstract

Meiotic recombination plays an essential role in the proper segregation of chromosomes at meiosis I in many sexually reproducing organisms. Meiotic recombination is initiated by the scheduled formation of genome-wide DNA double-strand breaks (DSBs). The timing of DSB formation is strictly controlled because unscheduled DSB formation is detrimental to genome integrity. Here, we investigated the role of DNA damage checkpoint mechanisms in the control of meiotic DSB formation using budding yeast. By using recombination defective mutants in which meiotic DSBs are not repaired, the effect of DNA damage checkpoint mutations on DSB formation was evaluated. The Tel1 (ATM) pathway mainly responds to unresected DSB ends, thus the *sae2* mutant background in which DSB ends remain intact was employed. On the other hand, the Mec1 (ATR) pathway is primarily used when DSB ends are resected, thus the *rad51 dmc1* double mutant background was employed in which highly resected DSBs accumulate. In order to separate the effect caused by unscheduled cell cycle progression, which is often associated with DNA damage checkpoint defects, we also employed the *ndt80* mutation which permanently arrests the meiotic cell cycle at prophase I. In the absence of Tel1, DSB formation was reduced in larger chromosomes (IV, VII, II and XI) whereas no significant reduction was found in smaller chromosomes (III and VI). On the other hand, the absence of Rad17 (a critical component of the ATR pathway) lead to an increase in DSB formation (chromosomes VII and II were tested). We propose that, within prophase I, the Tel1 pathway facilitates DSB formation, especially in bigger chromosomes, while the Mec1 pathway negatively regulates DSB formation. We also identified prophase I exit, which is under the control of the DNA damage checkpoint machinery, to be a critical event associated with down-regulating meiotic DSB formation.

## Introduction

Homologous recombination is essential for the accurate segregation of homologous chromosomes during meiosis [1]. Thus, there is a programmed induction of homologous recombination upon entry into meiosis. At the molecular level, the initiation of meiotic recombination is controlled by the genome-wide formation of DNA double-strand breaks (DSBs) [2].

The mechanism for controlling meiosis-specific DSB formation has been extensively characterized using budding yeast as a model organism. Meiotic DSBs are formed by the Spo11 protein, a meiosis-specific endonuclease that is homologous to type II topoisomerases [3]. DSB formation is coupled with the covalent linkage of Spo11 to the 5′-ends of DSBs. These Spo11 proteins need to be removed by endonucleolytic cleavage involving the Mre11/Rad50/Xrs2 complex and Sae2/Com1 for DSBs to be repaired through homologous recombination [4]. Once Spo11 is removed from the 5′ ends of DSBs, the 5′ ends receive further resection, leading to the exposure of 3′-ended single-stranded (ss) DNA strands [5]. These ssDNA strands are the substrates for homologous recombinases (i.e., RecA homologs, Rad51 and Dmc1 in yeast) that catalyze the homology searching and strand exchange reactions [6]. Virtually no meiotic DSBs are repaired in the absence of both Rad51 and Dmc1 [7].

Initiation of meiotic recombination needs to be coordinated with other events along the meiotic cell cycle. DSBs are most efficiently formed at the early stage of meiotic prophase I, and these DSBs finish being repaired toward the end of prophase I [6]. Thus, it is likely that there is a mechanism to control the activities of proteins involved in DSB formation during meiosis. The Mer2 protein is a target of such regulation. Mer2 is one of the essential ancillary factors of Spo11 and is regulated through phosphorylation by Cdc28 (budding yeast CDK1) and Dbf4-dependent Cdc7 kinase (DDK), two major kinases essential for cell cycle control; this phosphorylation is indispensable for meiotic DSB formation [8], [9], [10]. Furthermore, a recent study using mice and flies identified a link between DSB formation and DNA damage checkpoint mechanisms. ATM, a conserved protein kinase involved in triggering the DNA damage response, controls DSB formation such that DSB formation is down-regulated once DSBs are formed [11], [12].

Budding yeast has two major DNA damage checkpoint pathways that involve Mec1 and Tel1 respectively. Mec1 is the ortholog of ATR and is involved mainly in recognizing and responding to exposed ssDNA, whereas Tel1, the ortholog of ATM, responds to unprocessed DSB ends [13].

With budding yeast as a model organism, we investigated the possible involvement of DNA damage checkpoint mechanisms in the regulation of meiotic DSB formation. We found that DSB formation was reduced in larger chromosomes in the *sae2* mutant background. On the other hand, the absence of Rad17, a major component of the Mec1-dependent pathway, lead to an increase in DSB formation in the *rad51 dmc1* mutant background. Thus we propose that the Tel1 pathway facilitates DSB formation, especially in larger chromosomes, while the Mec1 pathway negatively regulates DSB formation. Furthermore, our results identified the transition from prophase I to metaphase I to be a critical event in down-regulating DSB formation.

## Results

### The *tel1* Mutation causes a Reduction in DSB Formation in Large Chromosomes

**Figure 1:**
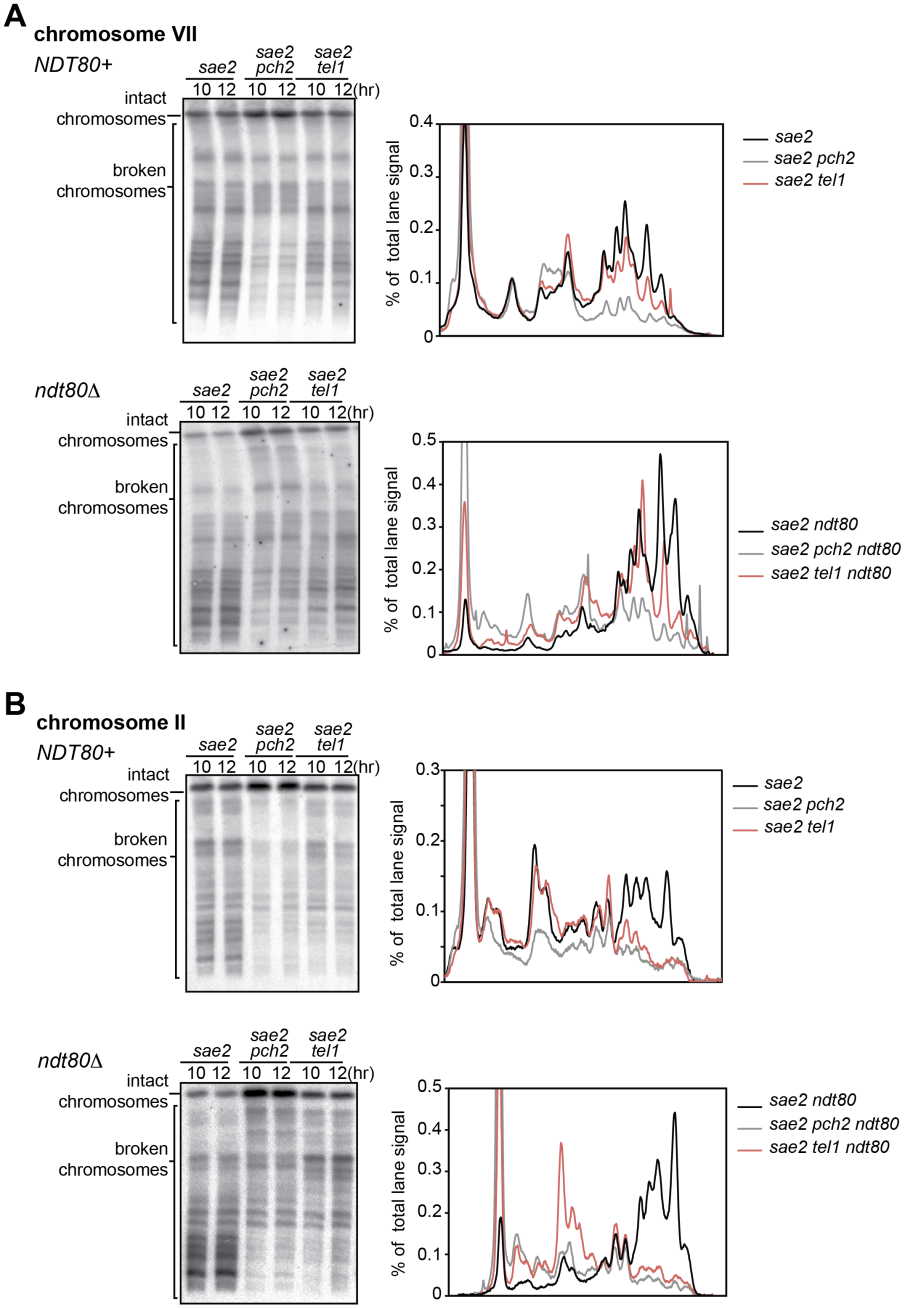
DSB formation is reduced by the *tel1* mutation and the effect is chromosome specific. Diploid *sae2*, *sae2 pch2* and *sae2 tel1* mutants in the *NDT80* positive background or the *ndt80* mutant background were introduced into meiosis and DSB formation was detected at indicated time points in chromosomes VII (A) and II (B). Lane profiles of 10 and 12 hours in each mutant background were normalized and averaged to obtain the profiles shown on the right. Cells from the same time course were used to examine both chromosomes VII and II. The Southern blot data used for *sae2* and *sae2 pch2* are the same as previously shown in [14].

**Figure 2:**
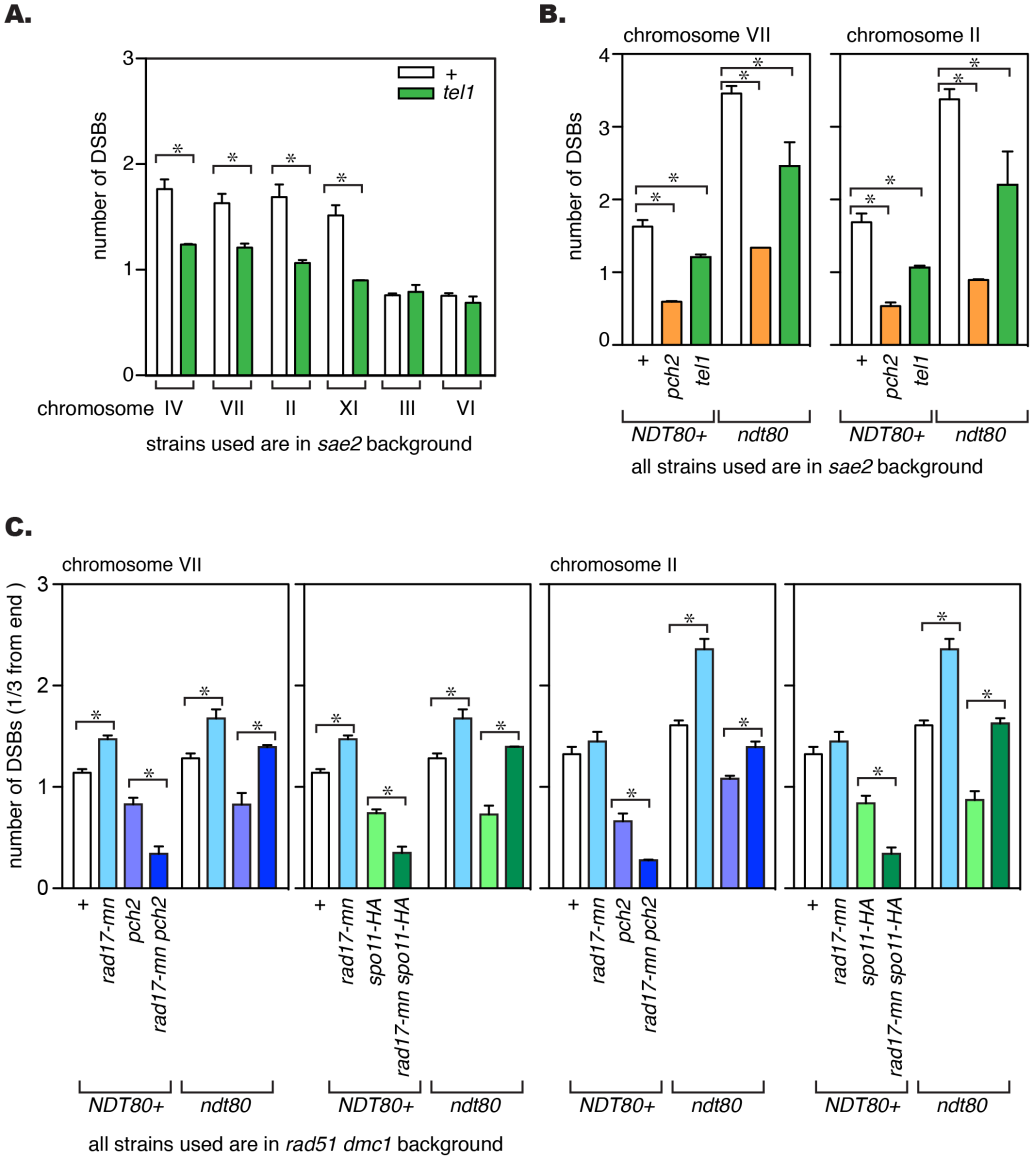
Quantitative analysis of meiotic DSB formation. DSB numbers were calculated using Southern blot data and the formula described in Materials and Methods. (A) The effect of the *tel1* mutation on DSB formation. (B) The *tel1* mutation reduces DSB formation in the absence of Ndt80 in chromosome VII and II. (C) *rad17-mn* effect on DSB formation in the presence of various mutations. A whole chromosome was used for DSB number calculation in the *sae2* mutant strains while one third of a chromosome was employed in the *rad51 dmc1* mutant strains (Materials and Methods). Error bars represent standard error. *, statistically significant (p<0.05, unpaired t-test). The actual data used to calculate DSB numbers are shown in Table S2.

The *sae2* mutant has often been used for evaluating the amount and distribution of meiotic DSBs. Under this condition, DSBs are not processed and the Tel1-dependent pathway is predominantly used for DNA damage responses [14]. To test the involvement of this pathway in DSB formation, the effect of the *tel1* mutation on DSB formation was examined in the *sae2* mutant background. The efficiency of DSB formation was evaluated per chromosome using pulsed-field gel electrophoresis (PFGE) followed by Southern blotting, with probes specifically recognizing an end of a particular chromosome [14]. We found that DSB formation was mildly affected in the absence of Tel1, with a more marked reduction in bigger chromosomes (IV, VII, II and XI) while DSB levels stayed similar in smaller chromosomes (VI and III) (Figure 1 and 2A). A similar chromosome size dependent effect was observed in the *pch2* mutant, although the effect caused by the *tel1* mutation is much milder (Figure 1 and 2A) [14]. The presence of unrepaired DSBs is monitored by the DNA damage checkpoint, which slows down/arrests the cell cycle [15]. DSB formation is supposedly a prophase I-specific event, thus the untimely cell cycle progression in the *tel1 sae2* double mutant could contribute to a reduction in DSB formation. To test this possibility, we took advantage of the *ndt80* mutation in which the meiotic cell cycle arrests permanently at the end of prophase I [16]. The effect of the *tel1* mutation on DSB formation was examined in the *sae2 ndt80* background. Overall, more DSBs were formed in the *ndt80* mutant background, suggesting that the exit from prophase I has a negative impact on DSB formation (Figure 1 and 2B). Under this condition, the absence of Tel1 caused a reduction in DSB formation in chromosome VII and II, just like it did in the *NDT80* positive strain (Figure 1 and 2B), suggesting that the mechanism responsible for a reduction in DSB formation in *tel1* cells is executed within prophase I.

### The Loss of Rad17 has a Positive and a Negative Effect on DSB Formation

Next we examined the possible involvement of the Mec1 pathway in DSB formation. We took advantage of the *rad51 dmc1* double mutant in which DSBs are not repaired at all and accumulate extensive 3′-tailed ssDNA at their ends. In this condition, the Mec1 pathway is primarily employed for damage response [17]. Rad17 is a critical component of the Mec1-dependent pathway, but, unlike Mec1, Rad17 is not essential. The impact of the loss of Rad17 on DSB formation was examined in the *rad51 dmc1* mutant background. During this procedure, we found that combining the *rad51* and *rad17* mutations compromises normal cell growth, which often causes inefficient entry into meiosis. To avoid this problem, we put the *RAD17* ORF under the control of the *CLB2* promoter, which down-regulates transcription of the downstream ORF in a meiosis-specific manner. This allele is called *rad17-mn* (meiotic null) hereafter. Unlike the *rad17* null mutant, *rad17-mn* showed resistance to methyl methanesulfonate, a DNA damaging agent, to levels comparable to that of wild type (Figure S1A). On the other hand, the *rad17-mn* diploid showed a substantial reduction in spore viability (∼50%). This level of spore viability is slightly higher than that of the *rad17* null mutant (∼35%) but much lower than the ∼100% spore viability seen in wild type (Figure S1B). The Rad17 protein was detected in vegetatively growing cells, while the amount of protein was already substantially reduced in the cells after being incubated in presporulation medium (time zero). Importantly, the amount of Rad17 was reduced to levels that are undetectable by western blotting for the duration of meiosis (Figure S1C). Furthermore, in the *rad51 dmc1* double mutant background, a robust induction of Cdc5, a land mark event for exit from pachytene, was seen when *rad17-mn* was introduced but not with wild-type *RAD17* (Figure S1D).

**Figure 3:**
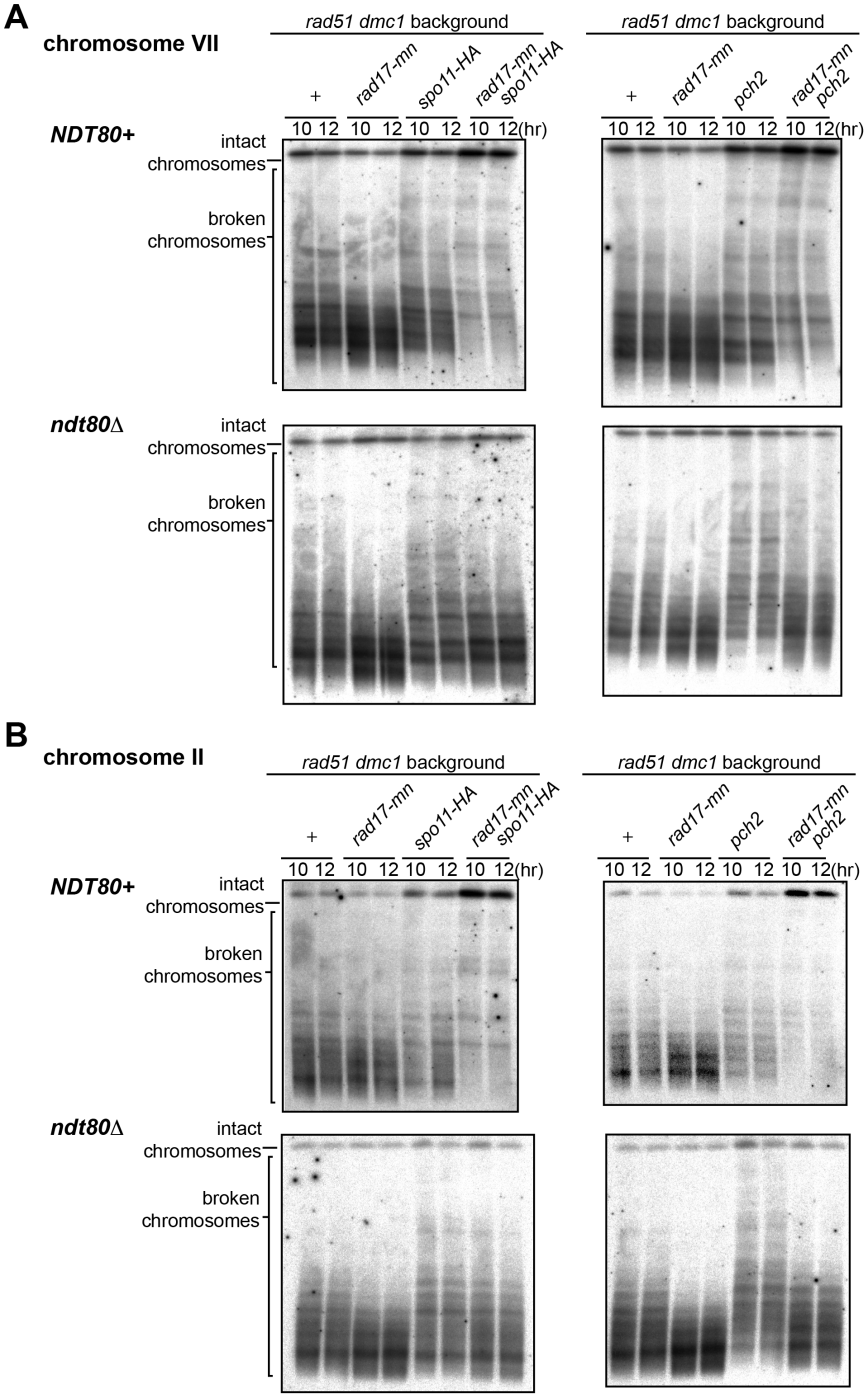
Positive and negative effect of the *rad17*-mutation on DSB formation. Diploid *rad51 dmc1* strains carrying various mutations as indicated, in the *NDT80* positive background or the *ndt80* mutant background, were introduced into meiosis and DSB formation was detected at indicated time points in chromosomes VII (A) and II (B).

**Figure 4:**
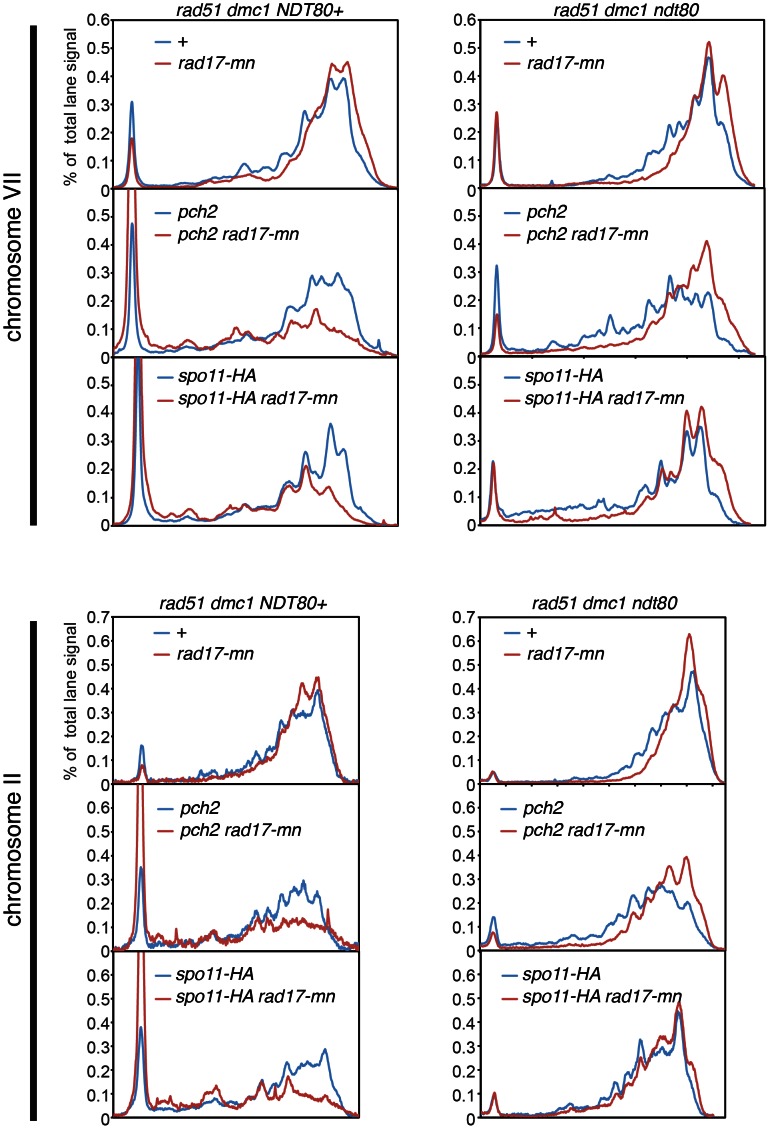
Comparison of lane profiles of broken meiotic chromosomes. Lane profiles of Southern blot signals shown in Figure 3 were compared between various mutants as indicated. Lane profiles of 10 and 12 hours in each mutant background were normalized and averaged to obtain the profiles shown.

The effect of the *rad17-mn* allele on DSB formation was examined in chromosomes VII and II using PFGE and Southern blotting. In the *rad17-mn* mutant, the overall size of broken chromosome fragments became smaller, indicating a mild increase in DSB formation (Figure 3). This difference is better demonstrated by comparing the lane profiles of broken chromosome fragments of *rad51 dmc1* and *rad51 dmc1 rad17-mn* mutants (Figure 4, red line versus blue line). Next the DSB formation efficiency of each mutant was quantitated. We noticed that in the *rad51 dmc1* double mutant strains, the efficiency of DSB formation is much higher than the *sae2* mutant, often leaving only a very small fraction of intact chromosomes. Since the quantitative analysis relies on the fraction of uncut chromosomes (Materials and Methods), the smaller the fraction of uncut chromosomes becomes, the more affected the calculated value becomes by other factors including a fraction of cells that did not go into meiosis and the quality of Southern blot (discussed in Materials and Methods). Thus, we analyzed part of each chromosome, from the end of a chromosome that a probe recognizes to one third of the total lane. This analysis showed that more DSBs are formed in the *rad17-mn* mutant in chromosome VII, which is statistically significant (Figure 2C). Although the averaged value for chromosome II in the *rad17-mn* is higher than that of the equivalent wild type *RAD17* strain, this difference turned out not to be statistically significant.

We previously showed that the *pch2* mutation reduces DSB formation [14]. Combining the *pch2* mutation with mutations in genes involved in the Mec1-dependent checkpoint pathway causes spore lethality [18]. Thus, we examined the combinational effect of the *pch2* mutation and *rad17-mn* allele on DSB formation. Also included in the analysis was *spo11-HA*, a hypomorphic allele of *SPO11* in which DSB formation is partially reduced [19]. In the *pch2* or *spo11-HA* mutant, DSB formation was mildly reduced as shown before, but the reduction was further pronounced when these mutations were combined with *rad17-mn* (Figures 2C, 3 and 4). Similar observations were made for both chromosomes VII and II.

In mutants where DNA damage checkpoint mechanisms are compromised, defects can be a direct consequence of a failure to activate the DNA damage checkpoint or an indirect result of unscheduled cell cycle progression. To distinguish these possibilities, the *ndt80* mutation was introduced in the strains described above (Figures 2C, 3 and 4). Overall, more DSBs are formed in the *ndt80* mutant background than in the *NDT80* positive counterparts (Figure 2C). The negative effect of the *pch2* or *spo11-HA* mutations on DSB formation was still observed in the *ndt80* background (Figure 2C). Strikingly, when *pch2* or *spo11-HA* was combined with *rad17-mn* in the *ndt80* mutant strains, DSB formation was more pronounced than in the corresponding *pch2* or *spo11-HA* mutants (Figure 2C). This result is the complete opposite of what was seen in the *NDT80* positive background. These findings suggest that the reduction in DSB formation seen in either *rad17-mn pch2* or *rad17-mn spo11-HA* in the *NDT80* positive background is caused by the unscheduled cell cycle progression associated with the checkpoint defect. Also, the more pronounced DSB formation seen in the *rad17-mn pch2* or *rad17-mn spo11-HA* mutants compared with their *pch2* or *spo11-HA* mutant counterparts further demonstrates the role of Rad17 as a negative regulator for meiotic DSB formation.

## Discussion

We investigated the possible roles of DNA damage checkpoint mechanisms in meiotic DSB formation using budding yeast. In order to quantitatively measure DSB formation, we employed genetic backgrounds in which DSB repair is defective; therefore the quantity of accumulated recombination intermediates is proportional to the amount of DSBs formed. However, the introduction of mutations in DNA damage checkpoint genes in such genetic backgrounds can cause a problem. The meiotic cell cycle in mutants defective in DSB repair is delayed/arrested in prophase I, and this phenomenon is suppressed when DNA damage checkpoint mechanisms are impaired. Since DSB formation usually occurs within prophase I, such unscheduled cell cycle progression itself can have a negative effect on DSB formation. By employing the *ndt80* mutation in which the meiotic cell cycle arrests at the late stage of prophase I, we separated the effect of cell cycle progression on DSB formation, which is associated with checkpoint mutants, from that seen within prophase I.

### Tel1 Facilitates DSB Formation in Large Chromosomes

Tel1 is the ATM ortholog in budding yeast and primarily responds to unprocessed DSBs, such as those that persist in the *sae2* mutant during meiosis. DSB formation was mildly decreased in the absence of Tel1 in large chromosomes, and this phenotype was not affected by the introduction of the *ndt80* mutation. These observations argue that Tel1 plays a positive role in meiotic DSB formation in these chromosomes. A similar trend was observed in the *pch2* mutant [14]. An interaction between Tel1 and Pch2 has previously been suggested [20], raising the possibility that the role of Tel1 and Pch2 in DSB formation might be related, although the *pch2* mutant shows a more substantial reduction in DSB formation.

The recruitment of Tel1 to DSB sites depends on the Mre11-Rad50-Xrs2 (MRX) complex [21], which is also essential for meiotic DSB formation in budding yeast [22]. Thus, the recruitment of the MRX complex to potential DSB sites before DSB formation is likely, suggesting a possible role for the MRX complex in recruiting Tel1 to chromosomes before DSB formation.

In ATM deficient mice, the total level of Spo11-oligonucleotide complexes is elevated [11]. The release of the complex is coupled with DSB formation, thus ATM negatively controls meiotic DSB formation. Also, in ATM-mutated flies, the level of phosphorylated histone H2AV, a marker for unrepaired DSBs, was elevated, leading to the suggestion that DSB formation is negatively regulated by ATM [12]. These trends are the opposite of what we have observed in budding yeast. However, it should be noted that our observation is based on the analysis using the *sae2* mutant background in which DSB ends are not resected, thus it is possible that such an effect is restricted to the *sae2* mutant or similar mutant backgrounds. Another possibility is that Tel1 (and possibly Mec1) also affects chromosome conformation or the rate of DSB processing, which can potentially lead to the differential accumulation of DSB markers such as histone H2AV or Spo11-linked oligonucleotides.

### Rad17 is a Negative Regulator of DSB Formation within Prophase I

Employing the *ndt80* mutant background made it possible to investigate the role of Rad17 in DSB formation independently of the cell cycle progression effect that is usually associated with a defect in the DNA damage checkpoint. In the *rad51 dmc1* double mutant background, DSB formation was pronounced in the absence of Rad17. A similar effect was seen when *pch2* or *spo11-HA* was combined with *rad17-mn* in the *ndt80* background. These results demonstrate the role of Rad17 in negatively regulating DSB formation. Rad17 is an indispensible component of the Mec1(ATR)-dependent DNA damage checkpoint pathway. ATR is recruited to ssDNA through its interaction with RPA and activated by interacting with the PCNA-like 9-1-1 complex consisting of Ddc1, Mec3 and Rad17 [23], [24]. Thus, the ATR pathway is likely to be in charge of responding to DSB formation to down-regulate DSB formation in budding yeast.

The difference in the usage of the ATM and ATR pathways in mice and budding yeast is interesting, given that ATM is primarily used for down-regulating DSB formation in mice. Once DSBs are formed, ATM is primarily used in responding to DSBs in mice whereas ATR (Mec1) is the major pathway in budding yeast. Thus, the apparent bias toward ATR utilization in budding yeast might reflect the overall usage preference to ATR in choosing a damage response pathway. After all, if meiotic DSBs, once formed, are processed to expose ssDNA in a relatively prompt manner, ATR would almost inevitably become the pathway of choice because ATM is less likely to be retained on processed DSB ends. On the other hand, in mice, it is possible that meiotic DSB ends are kept unprocessed for some time, which might allow ATM to respond to them, sending a negative feedback signal to the DSB forming mechanism.

### Exit from Prophase I Plays a Critical Role in Down-regulating DSB Formation

Our results further highlighted the importance of Ndt80 as a negative controller of DSB formation. First, more DSBs are formed in the *ndt80* mutants in general, in both *sae2* and *rad51 dmc1* mutant backgrounds, than their *NDT80* positive counterparts. Second, when DSB formation is compromised (i.e., *pch2* and *spo11-HA* mutations), the reduction in DSB formation is further exacerbated by a DNA damage checkpoint defect (i.e., *rad17* mutation), which is completely suppressed by arresting the cell cycle at prophase I (i.e., *ndt80* mutation). Thus, the synergistic effect between DSB formation inefficiency and a DNA damage checkpoint defect in the *NDT80* positive background is due to the Ndt80-dependent cell cycle progression.

Ndt80 is the master regulator that controls exit from prophase I and entry into metaphase I. Ndt80 is a downstream target of the DNA damage checkpoint mechanism during meiosis (recombination/pachytene checkpoint), which functions to coordinate homologous recombination (DSB repair) and cell cycle progression [25], [26]. Thus, we propose the decision to exit prophase I and enter metaphase I is highly associated with deactivating the DSB forming mechanism.

### Other Possible Mechanisms for Controlling Meiotic DSB Formation

The presence of unrepaired DSBs is sensed by DNA damage checkpoint mechanisms. In this work, we showed that Rad17 (and most likely the ATR pathway) is in charge of repressing DSB formation once DSBs are formed. Our results also suggest that DSB formation is shut off when cells exit prophase I. However, cells exit prophase I when the previously formed DSBs are repaired. This is contradictory because, as DSBs are repaired, the ATR-pathway becomes less active, possibly leading to reactivation of DSB formation. It is therefore likely that an unknown mechanism is responsible for gradually diminishing the DSB formation activity towards the end of prophase I. This mechanism may utilize the progress of homologous recombination as a temporal marker for prophase I. For example, the loading of proteins involved in later stages of homologous recombination, such as resolvases, and the formation of the synaptonemal complex can be exploited to serve such roles.

## Materials and Methods

### Yeast Strains

Genotypes of yeast strains are given in Table S1. All yeast strains used are isogenic derivatives of SK1. All markers were introduced by transformation and by genetic crosses between transformants and/or existing strains. The ORFs of *RAD51*, *DMC1*, *PCH2*, *SAE2, TEL1* were replaced with drug resistant markers by PCR mediated gene disruption [27]. To construct the *rad17-mn* allele, the promoter of *CLB2* was inserted immediately before the start codon of the *RAD17* ORF by PCR [28]. *rad51::URA3*, *spo11-HA*, *ndt80::LEU2* were previously described [5], [18], [29].

Strains used are: TBR5514, 5515, 5188, 6618, 6619 and 6620 in Figure 1; TBR6920, 6742, 6939, 6904, 6908, 6864, 6918, 6884, 6396, 6888, 6906 and 6862 in Figure 3; TBR3451, 6730 and 5696 in Figure S1A, 6621, 6749 and a diploid made of 5696 and 5698 in S1B, 6749 in S1C, and 6920 and 6742 in S1D.

### Meiotic Time Course and Detection of Meiotic DSBs

SK1 strains were introduced into meiosis as described previously with minor modifications [30]. Briefly, cells from a saturated culture in YPD supplemented with adenine (0.3 mM) and uracil (0.2 mM) were diluted in buffered YTA media (1% yeast extract, 2% tryptone, 1% potassium acetate, 50mM potassium phthalate) [31], and incubated for 12 hours. The pre-sporulation culture was washed once with water, and resuspended in 2% potassium acetate. Cells were harvested at appropriate time points and stored at −80°C until use.

Meiotic DSBs were detected as described previously with minor modifications [32]. Briefly, genomic DNA was prepared inside agarose plugs and separated on PFGE (120°, 14°C, 24 hours at 6 V/cm). Switching times applied are: 5 to 30 seconds for chromosome VI and III, and 20 to 60 seconds for the rest. Separated DNA was subjected to Southern blotting, with each chromosome visualized using radiolabeled probes annealing specifically to the chromosome. The radiolabeled membrane was imaged by a phosphoimager (Fuji, FLA5100). The obtained images were background-subtracted using AIDA (Raytest), an image analysis software, and the lane profiles were exported and further analyzed using Excel (Microsoft). Normalized lane profiles were obtained with each point divided by the total amount of signal per lane. 10 and 12 hour lane profiles were averaged to obtain the lane profiles presented. Probes for Southern blotting were previously described [14].

### Evaluation of DSB Formation Efficiency

Calculations to obtain the estimated DSBs on chromosomes used in Figure 2 were done as previously described [15], [33]. Briefly, the expected number of DSBs is obtained by
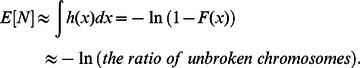



Based on this equation, E[N] solely relies on the signal ratio of unbroken chromosomes per total lane signal. This is an accurate estimate based on the assumption that 100% of cells enter into meiosis and the overall DSB distribution is not substantially affected by the introduced mutations. However, when the ratio of unbroken chromosomes becomes very small (<5%), the calculation is more easily affected from other factors such as a fraction of cells that did not go into meiosis and the quality of Southern blot. Thus, in the *rad51 dmc1* double mutant strains in which DSB formation is much more efficient than the *sae2* strains, we employed only one third of a chromosome for analysis, from the end of a chromosome that a probe recognizes to one third of the total lane length. The value equivalent to the ratio of unbroken chromosomes at this position is given by the signal ratio of the signal corresponding to the other two thirds of the lane including unbroken chromosomes per total lane signal. This way all the DSB numbers are widely dispersed within the range of 2.5 between the variety of the *rad51 dmc1* mutant strains (Figure 2 and Table S2).

## Supporting Information

Figure S1 **The** ***rad17-mn*** **allele reduces functionality of** ***RAD17*** **in a meiosis-specific manner.** (A) Vegetatively growing cultures of indicated strains were serially diluted and spotted on complete medium with or without methylmethane sulfonate (MMS). Two independent cultures were tested per genotype. (B) Diploid strains as indicated were sporulated and spore viability was measured by tetrad dissection. 40 tetrads were dissected per strain. (C) *rad17*-mn diploid cells before and after introduction into meiosis were examined for the production of the Rad17 protein by western blotting. Rad17 in the *rad17*-mn strain is tagged with the HA epitope, and thus can be detected with anti-HA antibodies. veg., vegetatively growing cells. (D) Indicated diploid strains were introduced into meiosis and the level of the Cdc5 protein, a marker for exit from the pachytene stage of prophase I, was examined by western blotting.
(TIF)

Table S1 **Yeast strains.**
(PDF)

Table S2 **Numbers used to calculate DSB amount in** **Figure 2** **.**
(PDF)
