# Supplementary figures and images for: Direct and Indirect Control of the Initiation of Meiotic Recombination by DNA Damage Checkpoint Mechanisms in Budding Yeast
Source: PLoS One. 2013 Jun 10;8(6):e65875. doi: 10.1371/journal.pone.0065875 (PMC3677890; doi:10.1371/journal.pone.0065875)

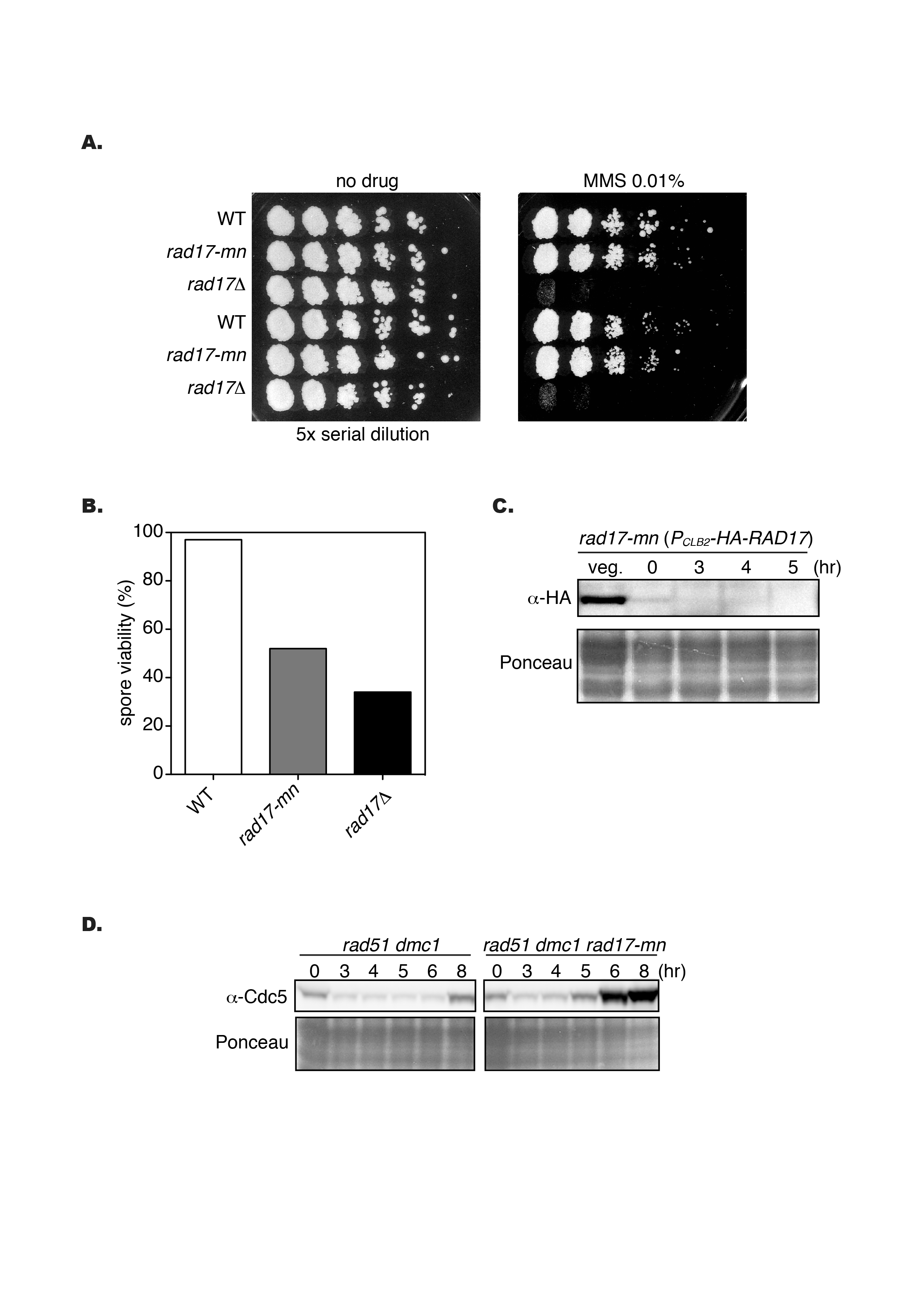

Supplement: Figure S1 — The rad17-mn allele reduces functionality of RAD17 in a meiosis-specific manner. (A) Vegetatively growing cultures of indicated strains were serially diluted and spotted on complete medium with or without methylmethane sulfonate (MMS). Two independent cultures were tested per genotype. (B) Diploid strains as indicated were sporulated and spore viability was measured by tetrad dissection. 40 tetrads were dissected per strain. (C) rad17-mn diploid cells before and after introduction into meiosis were examined for the production of the Rad17 protein by western blotting. Rad17 in the rad17-mn strain is tagged with the HA epitope, and thus can be detected with anti-HA antibodies. veg., vegetatively growing cells. (D) Indicated diploid strains were introduced into meiosis and the level of the Cdc5 protein, a marker for exit from the pachytene stage of prophase I, was examined by western blotting. (TIF) [file pone.0065875.s001.tif]
