# Supplementary material for: Direct and Indirect Control of the Initiation of Meiotic Recombination by DNA Damage Checkpoint Mechanisms in Budding Yeast
Source: PLoS One. 2013 Jun 10;8(6):e65875. doi: 10.1371/journal.pone.0065875 (PMC3677890; doi:10.1371/journal.pone.0065875)
Supplement: Table S1 — Yeast strains. (PDF) [file pone.0065875.s002.pdf]

Table S1. Yeast strains

Strain	Genotype
TBR5514	*MAT***a** *ho::hisG leu2::hisG ura3(Δ Sma-Pst) HIS4::LEU2-(NBam) sae2::KAN*<br>--<br>*MAT*α *ho::hisG leu2::hisG ura3(Δ Sma-Pst) his4X::LEU2-(NBam)-URA3 sae2::KAN*
TBR5515	TBR5514 but homozygous *pch2::HYG*
TBR5188	TBR5514 but homozygous *tel1::NAT*
TBR6618	*MAT***a** *ho::LYS2 lys2 ura3 leu2::hisG his3::hisG trp1::hisG sae2::KAN ndt80::LEU2*<br>--<br>*MAT*α *ho::LYS2 lys2 ura3 leu2::hisG his3::hisG trp1::hisG sae2::KAN ndt80::LEU2*
TBR6619	TBR6618 but homozygous *pch2::HYG*
TBR6620	TBR2065 but homozygous *tel1::NAT*
TBR6920	*MAT***a** *ho::LYS2 lys2 ura3 leu2::hisG his3::hisG trp1::hisG rad51::hisGURA3hisG dmc1:: NAT*<br>--<br>*MAT*α *ho::LYS2 lys2 ura3 leu2::hisG his3::hisG trp1::hisG rad51::hisGURA3hisG dmc1:: NAT*
TBR6742	TBR6920 but homozygous *KAN-*P_{CLB2}*-HA-RAD17*
TBR6939	TBR6920 but homozygous *spo11-HA::KAN*
TBR6904	TBR6920 but homozygous *KAN-*P_{CLB2}*-HA-RAD17 spo11-HA::KAN*
TBR6908	TBR6920 but homozygous *pch2::HYG*

TBR6864	TBR6920 but homozygous *KAN-*P_{CLB2}*-HA-RAD17 pch2::HYG*
TBR6918	*MAT***a** *ho::LYS2 lys2 ura3 leu2::hisG his3::hisG trp1::hisG rad51::hisGURA3hisG dmc1:: NAT ndt80::LEU2*<br>--<br>*MATα ho::LYS2 lys2 ura3 leu2::hisG his3::hisG trp1::hisG rad51::hisGURA3hisG dmc1:: NAT ndt80::LEU2*
TBR6884	TBR6918 but homozygous *KAN-*P_{CLB2}*-HA-RAD17*
TBR6396	TBR6918 but homozygous *spo11-HA::KAN*
TBR6888	TBR6918 but homozygous *KAN-*P_{CLB2}*-HA-RAD17 spo11-HA::KAN*
TBR6906	TBR6918 but homozygous *pch2::HYG*
TBR6862	TBR6918 but homozygous *KAN-*P_{CLB2}*-HA-RAD17 pch2::HYG*
TBR3451	*MAT***a** *ho::LYS2 lys2 ura3 leu2::hisG his3::hisG trp1::hisG*
TBR6730	TBR3451 but *KAN-*P_{CLB2}*-HA-RAD17*
TBR5696	TBR3451 but *rad17::NAT*
TBR5697	TBR3451 *MATα rad17::NAT*
TBR6621	*MAT***a** *ho::LYS2 lys2 ura3 leu2::hisG his3::hisG trp1::hisG*<br>---<br>*MATα ho::LYS2 lys2 ura3 leu2::hisG his3::hisG trp1::hisG*
TBR6749	TBR6921 but homozygous *KAN-*P_{CLB2}*-HA-RAD17*

All listed strains are SK1 derivatives.
