# Supplementary material for: Direct and Indirect Control of the Initiation of Meiotic Recombination by DNA Damage Checkpoint Mechanisms in Budding Yeast
Source: PLoS One. 2013 Jun 10;8(6):e65875. doi: 10.1371/journal.pone.0065875 (PMC3677890; doi:10.1371/journal.pone.0065875)
Supplement: Table S2 — Numbers used to calculate DSB amount in Figure 2 . (PDF) [file pone.0065875.s003.pdf]

Table S2. Numbers used to calculate DSB amount in Figure 2

A. Numbers used in Figure 2A

genotype	*sae2*											
	+	*tel1*	+	*tel1*	+	*tel1*	+	*tel1*	+	*tel1*	+	*tel1*
chromosome	4		7		2		11		3		6	
uncut/total	0.21	0.29	0.24	0.31	0.24	0.35	0.28	0.41	0.48	0.48	0.49	0.53
	0.17	0.29	0.18	0.29	0.17	0.34	0.24	0.41	0.49	0.42	0.44	0.47
	0.18		0.16		0.14		0.18		0.46		0.47	
	0.13		0.22		0.20		0.19		0.45		0.49	

B. Numbers used in Figure 2B

genotype	*sae2*											
	NDT80+						*ndt80*					
	+	*pch2*	*tel1*	+	*pch2*	*tel1*	+	*pch2*	*tel1*	+	*pch2*	*tel1*
chromosome	7			2			7			2		
uncut/total	0.24	0.55	0.31	0.24	0.61	0.35	0.04	0.26	0.12	0.05	0.40	0.18
	0.18	0.55	0.29	0.17	0.56	0.34	0.03	0.26	0.06	0.03	0.41	0.07
	0.16			0.14			0.03			0.04		
	0.22			0.20			0.03			0.02		

C. Numbers used in Figure 2C

genotype	*rad51 dmc1*											
	NDT80+											
	+	*rad17-mn*	*spo11-HA*	*rad17-mn spo11-HA*	*pch2*	*rad17-mn pch2*	+	*rad17-mn*	*spo11-HA*	*rad17-mn spo11-HA*	*pch2*	*rad17-mn pch2*
chromosome	7						2					
uncut/total	0.29	0.24	0.39	0.65	0.51	0.68	0.26	0.26	0.55	0.76	0.40	0.76
	0.33	0.24	0.39	0.85	0.43	0.59	0.23	0.30	0.48	0.76	0.46	0.67
	0.30	0.23	0.50	0.62	0.50	0.78	0.30	0.19				
	0.30	0.20	0.48	0.75	0.47	0.78		0.21				
	0.30	0.21										
	0.35	0.21										
	0.31	0.28										
	0.38	0.23										

genotype	*rad51 dmc1*											
	ndt80											
	+	*rad17-mn*	*spo11-HA*	*rad17-mn spo11-HA*	*pch2*	*rad17-mn pch2*	+	*rad17-mn*	*spo11-HA*	*rad17-mn spo11-HA*	*pch2*	*rad17-mn pch2*
chromosome	7						2					
uncut/total	0.26	0.24	0.49	0.25	0.53	0.25	0.22	0.11	0.35	0.26	0.45	0.21
	0.30	0.17	0.39	0.24	0.44	0.25	0.21	0.11	0.33	0.23	0.38	0.19
	0.25	0.16					0.18	0.08				
	0.30	0.18					0.20	0.08				
